# The burden of renal admissions in a tertiary Hospital in Sierra Leone

**DOI:** 10.1186/s12882-022-02806-7

**Published:** 2022-05-02

**Authors:** Joshua Coker, Onome Abiri, Obinna Jude Nwosu, Alhaji Gbla, Adetunji Wilson Taylor, Durodami Lisk

**Affiliations:** 1 Department of Medicine, Connaught Hospital, University of Sierra Leone Teaching Hospitals Complex, Freetown, Sierra Leone; 2 University of Sierra Leone, Freetown, Sierra Leone; 3 Pharmacy Board of Sierra Leone, Freetown, Sierra Leone

**Keywords:** Burden of renal admissions in Sierra Leone

## Abstract

**Background:**

The burden of both acute kidney injury and chronic kidney disease is on the rise globally. In sierra Leone, there has been no data on renal patients or admissions. This study intends to close this gap in knowledge and give preliminary data on the burden of renal disease in this country.

**Methods:**

The study was a retrospective review of the case notes of patients admitted at Connaught Hospital, Freetown over a 2 year period. Data extraction was done using a well- structured proforma.

**Results:**

A 2.7% renal admission burden was obtained; mean duration of hospital stay was 15.1 ± 14.7; mean age of patients was 47.2 ± 17.5 with a female preponderance. The common risk factors for chronic kidney disease were systemic hypertension (43%) and diabetes mellitus (24%). The common risk factors for acute kidney injury were sepsis (77%) and hypovolemia (15%). The in- hospital mortality rate was 47% and 73% were non-compliant with haemodialysis probably due to financial reasons.

**Conclusion:**

There is a significant burden of kidney disease in our environment, affecting mainly our young and middle-aged population. A rational approach is to embark on kidney disease prevention programs.

## Introduction

The impact of chronic kidney disease (CKD) on global health is escalating in terms of morbidity and mortality. An estimated global prevalence of chronic kidney disease was 9.1% in 2017, accounting for 697·5 million cases of all-stage CKD, with approximately 1·2 million persons dying from the disease [1]. In addition, there is a rising burden of non-communicable disease including kidney disease, systemic hypertension and diabetes mellitus in Africa and a corresponding rise in their related morbidity and mortality [2]. A systematic review and meta-analysis of articles published from 21 countries in sub-Saharan Africa revealed a 13.9% prevalence of chronic kidney disease [3]. However, there are no exact estimates of the prevalence of chronic kidney disease due to the lack of renal registries in many countries in sub-Saharan Africa [4]. The prevalent causes of end stage kidney disease in our environment are systemic hypertension and chronic glomerulonephritis and it affects mainly the young age group in contrast to industrialized countries where most of the patients are elderly [4]. In West Africa, the prevalence of chronic kidney disease is about 3.3% in Benin [5] and approximately 8–10% in Nigeria [6, 7]. These estimates were obtained from hospital studies which might reflect only the tip of the iceberg.

Prior to December 2016, Sierra Leone had no facility for renal replacement therapy in the form of haemodialysis. This means that all patients with end-stage renal disease or severe acute kidney injury requiring dialysis lost their lives. This describes the poor state of renal care in Sierra Leone and there is no previous data on hospitalized renal cases in Sierra Leone. This study aims to close this gap in knowledge and give preliminary data on admitted renal cases in Sierra Leone. This information might be useful for planning and improving renal care services in Sierra Leone.

## Methods

### Study setting

The study was conducted at the Connaught Hospital, University of Sierra Leone Teaching Hospitals Complex (USLTHC) situated in Freetown, the capital city of Sierra Leone with about 312 bed capacity. The hospital departments include surgery, internal medicine, pharmacy, nursing, radiology, laboratory, ophthalmology, oral health and ear, nose and throat.

### Study design and population

The study was a retrospective review of case notes of patients with renal impairment (acute or chronic) admitted at the cardio-renal ward, intensive care unit and other medical wards between November 2018 and October 2020.

### Inclusion/exclusion criteria

The study included all patients with renal disease (whether they require haemodialysis or not) who were ≥18 years old and who were admitted at the Connaught Hospital during the study period and whose case notes were retrievable. All patients below 18 years old as well as non- renal admissions were excluded.

### Data collection tool and procedure

Data collection was done using a structured proforma from the case notes of patients admitted at the cardio-renal ward, intensive care unit and other medical wards. The data collected included socio demographic characteristics, risk factors for chronic kidney disease, risk factors for acute kidney injury, common presenting symptoms of renal disease, examination findings, laboratory findings, duration of hospital stay and outcomes of renal admisisions. We defined renal admissions as any patient admitted with a serum Creatinine of ≥2 mg/dl or those admitted with a diagnosis of chronic kidney disease or acute kidney injury. Patients were classified as having chronic kidney disease based on the presence of symptoms of kidney disease lasting ≥3 months and 2 or more of the following: estimated GFR < 60 ml/min/1.73m^2^, presence of proteinuria or sonographic features like shrunken kidneys or loss of corticomedullary differentiation [8]. W**e** calculated their estimated GFR (Glomerular filtration rate) using the Chronic kidney disease epidemiology equation (CKD-EPI) based on their serum Creatinine. Patients were classified as acute kidney injury when the symptoms of kidney disease were < 4 weeks and at least one of the following: serum creatinine is > 2 mg/dl or urine output ≤0.3 ml/kg/hour [9]. We also obtained the total number of medical admissions during the study period in order to calculate the burden of renal admissions.

### Data management

All data collected were cross-checked and entered into a Microsoft Excel spreadsheet, and exported to statistical package for social sciences (SPSS) statistical software for analysis. Descriptive statistics, including frequencies, percentages, mean and standard deviations (SD) was used to summarize study variables/ Binary logistic regression was used to analyze the association between patients who had dialysis while on admission and their related mortality using odds ratio (OR) at 95% confidence level. A *P*-value of < 0.05 was considered statistically significant. Data was presented using tables and figures.

## Results

A total of 3741 admissions were made into the medical wards including the cardio renal ward and intensive care unit. Out of this, 100 were renal admissions(as stated in the methods section). This gives a 2.7% burden of renal admissions. The mean duration of hospital stay (days) for these patients was 15.1 ± 14.7; 51% were unemployed, 73% had no formal education, 4% were HIV positive and 73% were non–compliant with haemodialysis. The outcomes of renal admissions were as follows: 45% were discharged home, 47% died on admission, 2% were discharged against medical advice, 6% were re-admitted.

Majority of these patients (71%) were admitted with estimated GFR (eGFR) less than 15 mls/min/1.73m^2^ using CKD-EPI equation; 3% had EGFR 15-29mls/min/1.73m^2^; 5% had eGFR 30-44 ml/min/1.73m^2^; 8% had eGFR 45-59 ml/min/1.73m^2^;7% had eGFR 60-89 ml/min/1.73m^2^ and 6% had eGFR ≥90 ml/min/1.73m^2^.

Table 1 shows the socio-demographic characteristics of the patients. The mean age was 47.2 ± 17.5; a female preponderance of 57.0%; 77.2% of patients were < 60 years old; a peak age prevalence in the sixth (6th) decade. The commonest symptoms were bilateral leg swelling and decreased urine output.

**Table 1:** Shows the Socio-demographic characteristics of the patients and symptoms of patients with renal disease

Socio-Demographics	Frequency (f) *N =* 100	Percentage(%)
**Age (Years**		
**Mean age**	**47.2 ± 17.5**	
< 20	7	7
21-30	13	13
31-40	17	17
41-50	17	17
51-60	25	25
61-70	15	15
71-80	4	4
> 81	2	2
**Sex**		
Male	43	43
Female	**57**	57
**Symptoms at Presentation**		
Facial puffiness	5	5
Bilateral leg swelling	37	37
Haematuria	2	2
Exertional dyspnoea	17	17
Passage of Foamy urine	5	5
Urgency	1	1
Dysuria	3	3
Poor streaming of urine	8	8
Reduction in urine output	23	23

Table 2 shows the common risk factors for chronic kidney disease and the common risk factors for acute kidney injury among these patients. The commonest risk factor was systemic hypertension (43%), followed by diabetes mellitus (24%). The commonest risk factors for acute kidney injury were sepsis (77%) and hypovolemia (15%).

**Table 2:** Shows the risk factors for chronic kidney disease and acute kidney injury

Risk factors for CKD	Frequency *N =* 75	Percentages (%)
Systemic Hypertension	32	43
Diabetes Mellitus	18	24
Dyslipidemia	4	5
Obesity	7	9
Chronic Urinary Obstruction	1	1
Smoking	13	18
**Risk Factors for AKI**	Frequency *N =* 25	Percentage
Herbal Concoction	1	4
Dehydration/hypovolemia	4	15
Acute urinary obstruction	1	4
Sepsis (two or more criteria for SIRS with suspected focus of infection)	19	77

Table 3 shows other parameters of these patients such as mean and median blood pressure, packed cell volume, serum electrolytes, urinalysis findings and a summary of abdominopelvic ultrasound scan reports.

**Table 3:** Shows Baseline parameters of renal patients

Parameters	Mean ± SD	Median
Systolic Blood Pressure (mmHg)	140.84 ± 34.26	130
Diastolic blood pressure (mmHg)	87.24 ± 23.12	82
Estimated glomerular filtration rate (ml/min)	23.13 ± 33.62	10.5
Packed cell volume (%)	8.29 ± 2.57	7.8
Serum urea (mmol/L)	40.64 ± 30.68	28.3
Serum sodium (mmol/L)	142.40 ± 11.03	123.02
Serum potassium (mmol/L)	4.62 ± 1.42	2.02
Serum magnesium (mg/dl)	1.64 ± 1.43	1.5
Serum chloride (mg/dl)	101.39 ± 24.30	92.8
Serum calcium (mg/dl)	8.87 ± 6.70	6.7
Serum phosphorus (mg/dl)	12.13 ± 23.48	16.6
Serum creatinine (mg/dl)	9.8 ± 8.8	6.9
Random Blood Glucose (mmol/L)	8.81 ± 4.83	7.7
**Urinalysis (dipstick**) ***N =*** **56**		
**Proteinuria**	Frequency	Percentages
**1+**	11	20
**2++**	20	36
**3+++**	25	44
**Haematuria**		
**1+**	16	28
**2++**	24	42
**3+++**	16	30
**Leucocytes**		
**Trace**	28	50
**1+**	11	20
**2++**	10	17
**3+++**	7	13
**Abdominopelvic ultrasound** ***N =*** **25**	Frequency	Percentages
Parenchymal Renal Disease	18	72
Ascites	2	8
Normal Ultrasound	3	12
Polycystic Kidney Disease	1	4
Urinary Bladder Mass	1	4

Table 4 describes the relationship between having dialysis while on admission and mortality during admission.

**Table 4:** Shows the relationship between patients who had dialysis while on admission and their related mortality

Dialysis	Mortality *N =* 47		*p-*value	OR (95%CI)
	Yes	No	0.04	2.16 (0.93-5.03)
Yes	10 (18.2%)	23 (32.4%)		
No	45 (81.8%)	48 (67.6%)		

## Discussion

The summary of findings from this study reveals a 2.7% admission burden, young age of patients affected with kidney disease, female preponderance, high level of unemployment among patients with kidney disease and the relationship between dialysis and mortality. The findings also highlighted systemic hypertension, diabetes mellitus as the commonest underlying risk factor for chronic kidney disease with sepsis and hypovolemia as the commonest risk factors for acute kidney injury.

A 2.7% admission burden might be an understatement as many people with kidney disease are asymptomatic or are residing in hard to reach areas. Also, the lack of a formidable referral system and facilities for conducting renal function tests in many primary and secondary health facilities are other contributory factors. In addition, this was a single center hospital-based study and so data collected might just be the tip of the iceberg. In another study, reasons cited for understating the actual burden of chronic kidney disease were patients remaining undiagnosed or finding solace in spiritual or traditional healing [10]. Studies done in other countries in sub Saharan Africa suggest 2- 5% of medical admissions in their respective tertiary hospitals in South Africa and Ghana [11, 12]. In the Ghanaian study, a 5% renal admission burden (mainly end stage renal disease) was obtained over an 8-month review period [12]. The mortality among end stage renal disease patients was documented as 27.1% [12]. Another study done in Nigeria suggested an increased burden of end-stage kidney disease patients and a high attrition rate after commencing dialysis due to financial reasons [13]. A four-year retrospective study done in Southern Nigeria revealed a 15.4% admission burden, while a 10 year retrospective review of renal admission done in Western Nigeria revealed a 10% admission burden [14, 15].

Many of our patients present with an estimated glomerular filtration rate less than 30mls/min/1.73m^2^ (stage IV and V). This suggests a relatively advanced renal failure and some may require renal replacement therapy in the form of dialysis. In addition, they are often anaemic and may require blood transfusion. This tends to delay the commencement of haemodialysis and extend their stay in hospital. The mean hospital stay for these patients is about 2 weeks. A summary of their laboratory values also suggest a mean occurrence of hypocalcaemia and hyperphosphatemia suggesting evidence of mineral bone disease. Urinalysis findings revealed a frequent occurrence of proteinuria (nephrotic and sub-nephrotic) and haematuria suggesting ongoing renal insults. The most frequent abdominopelvic ultrasound findings were renal parenchymal changes described as increased parenchymal echogenicity, loss of corticomedullary differentiation and shrunken kidneys suggesting longstanding renal injury. The commonest symptoms at presentation for the renal patients are bilateral leg swelling, reduction in urine output and exertional dyspnoea. These are well known symptoms of advanced renal failure. This late presentation leads to many unplanned dialysis sessions. Such patients will commence dialysis without adequate time for education on kidney disease and other options of renal replacement therapy as well as fashioning of an arteriovenous fistula. Late presentation is a major contributor to early mortality among haemodialysis patients [16, 17]. High rates of unemployment, expensive health care services, use of alternative treatments like spiritualists and traditional healers and the lack of regular screening for CKD and inadequate nephrology service may also contribute to late presentation [4].

The mean age of patients was less than 50 years old. A vast majority of the patients were less than 60 years old. There is a slight female preponderance as well as a high rate of unemployment among our patients. The young age at presentation of many of our patients may be due to a high rate of infections (especially bacterial and parasitic) in our environment which might affect the kidneys resulting in post-infectious glomerulonephritis and subsequently chronic kidney disease. The high rate of unemployment among our patients would suggest that many of them cannot afford to pay for their haemodialysis treatment or to buy drugs like erythropoietin, parenteral iron, phosphate binders or calcitriol. This often results in high morbidity and mortality among our patients. The major risk factors for chronic kidney disease were systemic hypertension and diabetes mellitus. The lack of data on chronic glomerulonephritis might have been due to documentation bias. However, we can say that the burden attributable to systemic hypertension will also include those with chronic glomerulonephritis as many of our patients are in the young age group.

Prior to 2021, the unavailability of kidney biopsy needles and other factors relating to human resource made kidney biopsy a remote possibility. Even with the use of kidney biopsy and the presence of Pathologists, making a tissue diagnosis of kidney disease is still limited by lack of special stains and absence of immunofluorescence and electron microscopy. Nevertheless, inferences are made from the Haematoxylin & Eosin stained specimen, clinical history and laboratory results so that some patients can be started on immunosuppresives or steroids. Some of the common histopathology seen so far are suggestive but not conclusive of minimal change disease, focal segmental glomerulosclerosis, focal mesangial proliferation and interstitial nephritis.

Sociodemographic factors may impact the effect of established risk factors on the development of kidney disease [18]. These factors may affect health through several means including inadequate access to preventive health care for screening and early detection of diseases and paucity of funds [19]. This may result in inadequate control of major risk factors of chronic kidney disease such as hypertension and diabetes mellitus [19]. Furthermore, women may be more vulnerable due to lower income and more unemployment [19]. In addition, women have additional risk factors for kidney disease like undiagnosed autoimmune disease, pregnancy-related acute kidney injury [20].

The major risk factors for acute kidney injury (AKI) were sepsis and hypovolemia. The high rate of both bacterial and parasitic infections in our locality, the poor health- seeking behavior of our patients which include late presentation to hospitals, use of alternative medicine might be contributory. In addition, the probable high rate of medical malpractice, antimicrobial resistance, increased incidence of water-borne diseases and many people living in unsanitary conditions may play a role. A single-center study done in Sudan also highlighted similar risk factors for acute kidney injury [21]. Globally, acute kidney injury is known to affect about 13 million persons, with 85% of those affected residing in developing countries [22].

It was difficult to apply the serum creatinine-based Kidney Disease Improving global outcomes (KDIGO) definition for acute kidney injury because many of our patients present late and cannot afford to do serum creatinine more than once [9]. A critical limitation of the KDIGO definition is that it requires a prior knowledge of the patient’s baseline creatinine. For many of the patients admitted for AKI in our hospital, there is no record of their baseline serum creatinine and an absolute rise in serum creatinine of greater than 0.3 mg/dl cannot be demonstrated for reasons cited above.

Many of our patients required renal replacement therapy while on admission. Patients who could not pay for dialysis died while on admission. Dialysis clearly reduces the in-hospital mortality of patients admitted with kidney disease (OR > 2; *P* < 0.05). The overall mortality rate among our patients was a little lower than 50%. Major contributors to patients not accessing haemodialysis include prohibitive costs, few dialysis units (located usually in cities) and shortage of skilled workers [23]. The mortality rates for non-renal admissions like stroke and HIV in our hospital are 39.5 and 30.1% respectively [24, 25]. This high mortality may be attributable to local health-care challenges, the description of which is beyond the scope of this article.

The admission burden of mainly unemployed young patients poses serious issues bordering around affordability of care even though affordability was not directly assessed in this study. The high mortality, especially among the non-dialyzed also suggest the role financial affordability plays in kidney care. Going forward, there is a need for screening programs directed at the major risk factors for CKD and AKI as earlier documented. Such a program should focus on early diagnosis, availability and compliance to drugs, access to care, regular follow-up, meeting treatment targets. Studies have documented that a well-executed prevention program will help to save lives, create major health gains and improving health equity by preventing end stage kidney disease [26]. The International Society of Nephrology (ISN) ‘0 by 25’ initiative advocates the reduction of preventable deaths related to AKI in the world [27]. Achieving this feat requires a concerted effort between government, non-governmental organizations (NGOs} and private partners working together to prevent deaths from AKI [28]. Such strategies will include raising awareness to issues such as infection prevention and control, early diagnosis and treatment of infections, appropriate management of diarrhoeal diseases and vomiting.

There is urgent need for government, private partners and NGOs to provide financial support to patients with kidney disease in order to improve equity to kidney health. There is also a desperate need to improve renal diagnostic facilities in Sierra Leone and also to embark on intensive renal prevention programs.

## Conclusion

There is a significant burden of kidney disease in our environment, affecting mainly the young and middle-aged population. Therefore, a plausible approach to improving this situation is to embark on prevention programs. The preventive approach is a cheaper option not only for Sierra Leone but also other countries in sub-Saharan Africa.

## Abbreviations

AKI: Acute kidney injury
CKD: Chronic kidney disease
CKD-EPI: Chronic kidney disease epidemiology equation
DAMA: Discharge against medical advice
eGFR: estimated glomerular filtration rate
GFR: Glomerular filtration rate
HIV: Human immunodeficiency virus
ISN: International Society of Nephrology
KDIGO: Kidney Disease Improving Global Outcomes
SPSS: Statistical package for social sciences
USLTHC: University of Sierra Leone Teaching Hospitals Complex
